# Analysis of Long Non-Coding RNA and mRNA Expression Profiling in Immature and Mature Bovine (*Bos taurus*) Testes

**DOI:** 10.3389/fgene.2019.00646

**Published:** 2019-07-05

**Authors:** Yuan Gao, Shipeng Li, Zhenyu Lai, Zihui Zhou, Fei Wu, Yongzhen Huang, Xianyong Lan, Chuzhao Lei, Hong Chen, Ruihua Dang

**Affiliations:** Key Laboratory of Animal Genetics, Breeding and Reproduction of Shaanxi Province, College of Animal Science and Technology, Northwest A&F University, Yangling, China

**Keywords:** cattle, lncRNAs, RNA-Seq, testis development, spermatogenesis

## Abstract

Testis development and spermatogenesis are strictly regulated by numbers of genes and non-coding genes. However, long non-coding RNAs (lncRNAs) as key regulators in multitudinous biological processes have not been systematically identified in bovine testes during sexual maturation. In this study, we comprehensively analyzed lncRNA and mRNA expression profiling of six bovine testes at 3 days after birth and 13 months by RNA sequencing. 23,735 lncRNAs and 22,118 mRNAs were identified, in which 540 lncRNAs (*P*-value < 0.05) and 3,525 mRNAs (*P*-adjust < 0.05) were significantly differentially expressed (DE) between two stages. Correspondingly, the results of RT-qPCR analysis showed well correlation with the transcriptome data. Moreover, GO and KEGG enrichment analyses showed that DE genes and target genes of DE lncRNAs were enriched in spermatogenesis. Furthermore, we constructed lncRNA–gene interaction networks; consequently, 15 DE lncRNAs and 12 *cis*-target genes were involved. The target genes (*SPATA16*, *TCF21*, *ZPBP*, *PACRG*, *ATP8B3*, *COMP*, *ACE*, and *OSBP2*) were found associated with bovine sexual maturation. In addition, the expression of lncRNAs and *cis*-target genes was detected in bovine Leydig cells, Sertoli cells, and spermatogonia. Our study identified and analyzed lncRNAs and mRNAs in testis tissues, suggesting that lncRNAs may regulate testis development and spermatogenesis. Our findings provided new insights for further investigation of biological function in bovine lncRNA.

## Introduction

The mammalian testis has crucial effects on male reproduction since is the site where spermatogenesis occurs (Griswold, 2016). Mammalian spermatogenesis comprises three continuous stages: self-renewal of mitotic proliferation spermatogonia, meiotic division of spermatocytes, and post-meiotic differentiation of haploid spermatids (Hecht, 1998). These three distinguishable events are under strict regulation by stage-specifically expressed genes at transcription and post-transcription process (Card et al., 2013; Djureinovic et al., 2014). More than 15,000 genes are expressed in the testis (Ramskold et al., 2009). In addition, this organ expresses more complex transcriptomes in comparison with other tissues (Soumillon et al., 2013). Rapid advancement has demonstrated that non-coding RNAs were emerging as important regulators of gene expression at post-transcription in spermatogenesis, such as microRNAs (miRNAs), long non-coding RNAs (lncRNAs), circular RNAs (circRNAs), and Piwi-interacting RNA (piRNAs) (Mukherjee et al., 2014; Robles et al., 2017; Gao et al., 2018; Qiu et al., 2018).

Studies have demonstrated that lncRNAs with multi exons and more than 200 nucleotides (nt) in length as their distinguishing feature play a key role in regulating gene expression in various biological processes, such as organ development (Luk et al., 2014; Paralkar et al., 2014; Zhao et al., 2015), X inactivation (Zhao et al., 2008; Bischoff et al., 2013), and genomic imprinting (Sleutels et al., 2002). Recently, numerous lncRNAs have been revealed that exhibited tissue-specific and developmental stage-specific expression (Koufariotis et al., 2015; Zhu et al., 2016). Some lncRNAs have been identified in different stages of testes development and spermatogenesis in mouse (8,265 lncRNAs and 18,563 mRNAs) (Sun et al., 2013), Drosophila (128 testis-specific lncRNAs) (Wen et al., 2016), pig (15,528 lncRNAs) (Ran et al., 2016), chicken (2,597 lncRNAs and 17,690 mRNAs) (Liu et al., 2017), and sheep (6,460 lncRNAs and 42,300 mRNAs) (Zhang et al., 2017). Some of them have certain regulatory function in testis development and spermatogenesis, such as HongrES2 (Ni et al., 2011), Tsx (Testis-specific X-linked) (Anguera et al., 2011), Dmrt1 (Dmrt1-related gene) (Zhang et al., 2010), and Mrh1 (meiotic recombination hot spot locus) (Arun et al., 2012). Knocking out of Tsx increased the apoptosis of pachytene spermatocytes in mice (Anguera et al., 2011). The interaction of Mrhl and its protein partner Ddx5/p68 negatively regulated Wnt signaling in mouse spermatogonial cells (Arun et al., 2012). Hence, we assume that bovine lncRNAs are also involved in bovine testis development. Moreover, a number of studies have reported putative bovine lncRNAs identified in early embryo (Caballero et al., 2014), skin (Weikard et al., 2013), mammary glands (Tong et al., 2017), skeletal muscle (Sun et al., 2016), and 18 cow tissues (Koufariotis et al., 2015). However, lncRNAs as key regulators in multitudinous biological processes have not been systematically identified in bovine testes during sexual maturation. Therefore, this study was carried out to further explore the regulation of lncRNA on development of bovine testis.

In this study, we comprehensively analyze the lncRNA and messenger RNA (mRNA) expression profiles of Angus cattle (*Bos taurus*) at two representative stages [3 days after birth (neonatal, pre-sex maturation) and 13 months (mature, post-sex maturation)] by constructing six RNA sequencing (RNA-Seq) libraries and sequencing. The aims of this study were to identify and feature lncRNAs in bovine testes and to detect key lncRNAs that related to testis development. This is the first study to systematically identify lncRNAs in bovine testes during postnatal development. Our data will provide a basis for exploiting their function of bovine testis development and spermatogenesis.

## Materials and Methods

### Animals and Sample Collection

Whole six testes from Angus bulls were collected from Shaanxi Kingbull Livestock Co., Ltd. (Baoji, China). Based on the time of sexual maturation (Lunstra, 1982), three Angus bulls were sacrificed at each of the two stages: 3 days after birth (neonatal, pre-sex-maturation) and 13 months (mature, post-sex maturation). The six Angus bulls were euthanized and testicular samples were dissected, while immediately frozen in liquid nitrogen until use. The data about volume of ejaculate, fresh sperm motility, and sperm concentration from mature bovine samples were shown in **Table S15**.

### Hematoxylin and Eosin Staining

Cattle testes tissues were washed with 0.9% saline, fixed in 4% formaldehyde for 48 h, and processed using routine histological methods (Fischer et al., 2008), and 6-μm sections were stained with hematoxylin–eosin. The morphology of testicular tissues was analyzed by light microscopy.

### RNA Isolation

According to the manufacturer’s protocol, total RNAs were extracted from testis tissue using Trizol reagent (Takara, Beijing, China) and then sent to Novogene (Beijing Novogene Corporation, China) for sequencing. RNA degradation and contamination were detected on 1% agarose gels. The purity of RNA samples was checked using the NanoPhotometer^®^ spectrophotometer (IMPLEN, CA, USA). The RNA concentration was conducted with Qubit^®^ RNA Assay Kit in Qubit^®^ 2.0 Fluorometer (Life Technologies, CA, USA). The RNA integrity was evaluated by the RNA Nano 6000 Assay Kit of the Bioanalyzer 2100 system (Agilent Technologies, CA, USA).

### Library Preparation for lncRNA Sequencing

Each RNA sample was the amount of 3 μg and was added as input material for the RNA sample preparations. Epicenter Ribo-zero^™^ rRNA Removal Kit (Epicentre, USA) was applied for removing ribosomal RNA. Sequencing libraries were generated using the rRNA-depleted RNA by NEBNext^®^ Ultra^™^ Directional RNA Library Prep Kit for Illumina^®^ (NEB, USA) following the manufacturer’s recommendations. Fragmented RNA with the average length was approximately 200 bp, then synthesized, and purified cDNA by reverse transcription. Products were purified (AMPure XP system) after library quality was assessed on the Agilent Bioanalyzer 2100 system. The clustering of the index-coded samples was performed on a cBot Cluster Generation System using TruSeq PE Cluster Kit v3-cBot-HS (Illumina). After cluster generation, the libraries were sequenced on an Illumina Hiseq 4000 platform, and 150 bp paired-end reads were generated.

### Alignment of RNA-Seq Reads and Assembly of Transcripts

Firstly, raw data were filtered for the purpose of removing low-quality reads. Then, paired-end clean reads were aligned to the reference genome (*B. taurus UMD3.1*) using HISAT2 (v2.0.4) and using bowtie2 (v2.2.8) to build the index of the reference genome (Langmead and Salzberg, 2012). The mapped reads of each sample were assembled by StringTie (v1.3.1) (Pertea et al., 2016) in a reference-based approach. Finally, assembled transcripts were annotated by Cuffcompare program from the Cufflinks package.

### Identification of Putative lncRNA

Putative lncRNAs were found with screening unknown transcripts. In order to reduce the rates of false positive, assembled transcripts were filtrated to gain putative lncRNAs by the following steps: 1) Transcripts with one exon were removed. 2) Transcripts with lengths above 200 nt were selected. 3) To remove the transcripts that overlap with the exon region of the database annotation using Cuffcompare, the annotated lncRNAs in database were used in further analysis. 4) The transcripts with low expression levels (FPKM < 0.5) were discarded (FPKM of a single exon transcript < 2). 5) Protein coding potency of transcripts was calculated by Coding-Non-Coding-Index (CNCI) (Sun et al., 2013), coded potential calculator (CPC) (Kong et al., 2007), and Pfam-scan (Bateman et al., 2002; Finn et al., 2014). Using these three tools, all transcripts predicted with coding potential were filtered out, and those without coding potential were our putative lncRNAs. Besides, the transcripts with uncertain coding potential were defined as transcripts of uncertain coding potential (TUPC). In addition, the different types of lncRNAs were selected using cuffcompare.

### Differential Expression Analysis

The gene expression level was estimated using the FPKMs value. Cuffdiff (v2.1.1) was used to calculate FPKMs of lncRNAs, TUPC, and mRNAs (Trapnell et al., 2010). Then, the statistically significant differentially expressed genes were obtained by a *P*-value <0.05 or *P*-adjust <0.05 using Ballgown (Frazee et al., 2015).

### Target Genes Prediction

Differentially expressed lncRNAs were chosen for predicting target genes. Cis role is lncRNA acting on neighboring of target genes. We searched among 100k upstream and downstream of lncRNA to find coding genes and then analyzed their function (Ghanbarian and Hurst, 2015). Trans role is lncRNA to interact with target genes by expression level. The expressed correlation was calculated between lncRNAs and coding genes with custom scripts, and a Pearson correlation coefficient >0.95 identified target genes (Liao et al., 2011).

### GO Terms and KEGG Pathway Enrichment.

Gene Ontology (GO) enrichment analysis of differentially expressed genes or lncRNA target genes was implemented by the GOseq R package, in which gene length bias was corrected (Young et al., 2010). GO terms with corrected *P*-value less than 0.05 were considered significantly enriched by differential expressed genes. The statistical enrichment of differential expression genes or lncRNA target genes was tested by using KOBAS software in Kyoto Encyclopedia of Genes and Genomes (KEGG) pathways (Mao et al., 2005).

### Experimental Validation of mRNAs and lncRNAs

To confirm individual mRNA and lncRNA, RNA was reverse transcribed using PrimeScript^™^ RT reagent kit with gDNA Eraser (Perfect Real Time) (Takara, Beijing, China) with random hexamers according to the manufacturer’s protocol. RT-qPCR was performed on a BioRad CFX96 machine using ChamQ^™^ SYBR qPCR Master Mix (Vazyme, Nanjing, China). Each 20 μl real-time RT-qPCR reaction included 10 μl 2x ChamQ SYBR qPCR Master Mix, 0.4 μl primer, 2 μl cDNA, and 7.2 μl ddH_2_O. PCR conditions consisted of 1 cycle at 95°C for 30 s, followed by 40 cycles at 95°C for 10 s, 60°C for 30 s. The relative expression level was calculated using the 2^−ΔΔCt^ method with β-actin as the control. The primer details were shown in **Table S13**.

### Cell Isolation and qRT-PCR Verification

Leydig cells and Sertoli cells were isolated from neonatal bovine testes. Leydig cells were isolated using collagenase IV (Sigma) (Yu et al., 2017), and Sertoli cells were isolated by the two-step enzymatic digestion method (Zhang et al., 2013). After 4–6 h of culture, Sertoli cells spontaneously adhered to the culture surface and non-adhering cells were removed. These two types of cells were cultured in DMEM/F12 (HyClone), supplemented with 10% FBS (Gibco), 100 U/ml penicillin, and 100 μg/ml streptomycin (HyClone), at 37°C in a 5% CO_2_ atmosphere. The Bovine mGSCs (male germline stem cells) were derived from an immortalized cell line (Lei et al., 2017). The cells were harvested for RT-qPCR, and the primer details were shown in **Table S14**.

### Statistical Analysis

All data were analyzed in GraphPad Prism 6 software (GraphPad Software, La Joya, CA, USA). The qPCR data are shown as the mean ± the standard deviation. Each group contains three biological replicates with each measurement repeated thrice. The normally distributed data were analyzed using Student’s *t*-test, and the non-normally distributed data were analyzed using Mann–Whitney *U*-test.

## Results

### Morphology of Testis Tissues

**Figure 1:**
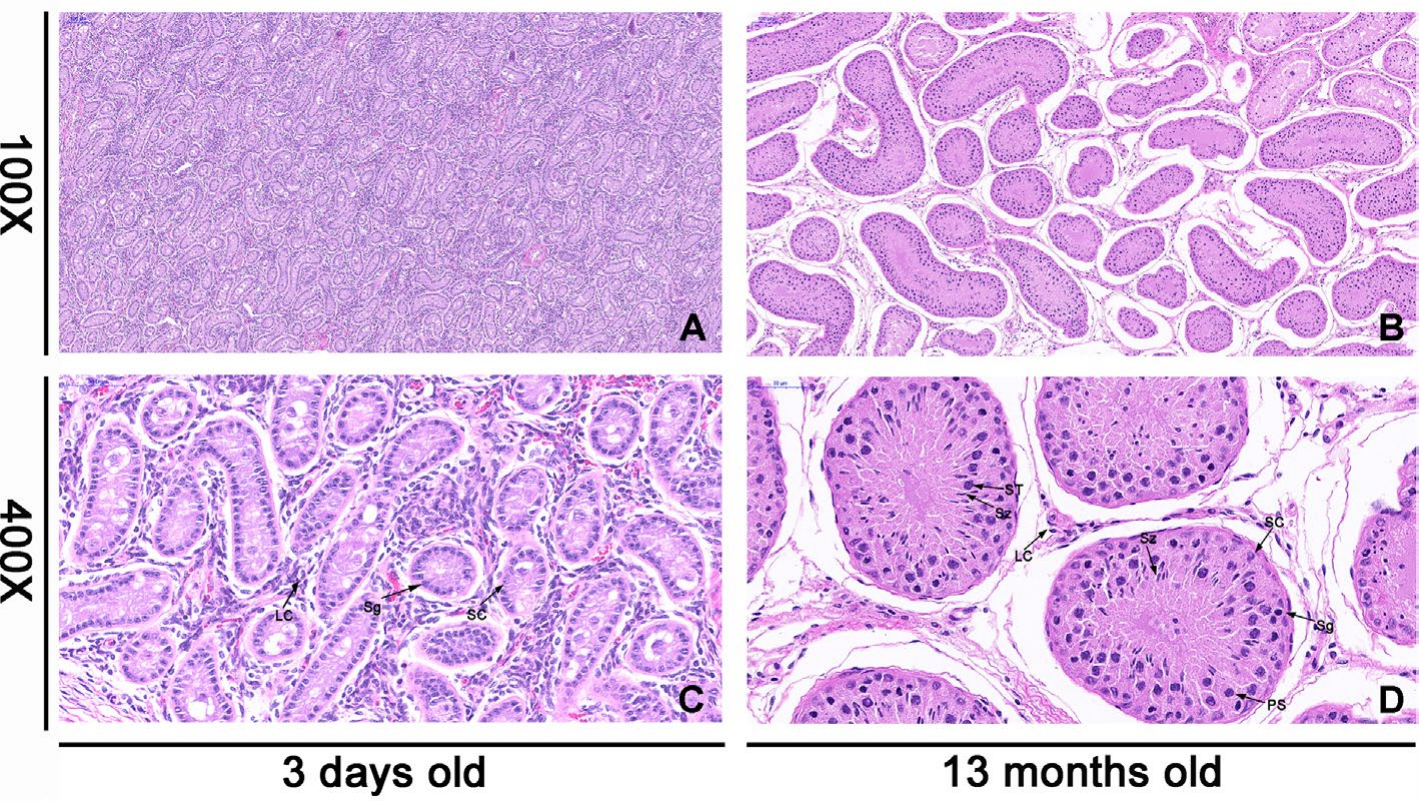
Histologic observations of cattle testis tissues at 3 days old (neonatal) and 13 months old (mature). The morphology of each sample was determined by light microscopy. The testicular tissues were observed under microscope at 100× and 400× magnification. a and c were the morphology of neonatal testicular tissue, while b and d were the morphology of mature testicular tissue. Panels **(A)** and **(B)** are in the same magnification. Bar = 100 μm. Panels **(C)** and **(D)** are in the same magnification. Bar = 50 μm. The black arrow shows different cell types. SC, Sertoli cell; LC, Leydig cell; Sg, spermatogonia; PS, primary spermatocyte; ST, spermatid; Sz, sperm.

As shown in **Figure 1**, the morphology of the testis showed significant difference at different development stages. The diameter of seminiferous tubules increased with age, whereas the interstitial connective tissue of mature testis was larger than that of the neonatal testis (**Figures 1A, B**). By contrast, the numbers of spermatogonia, sperm, and Sertoli cells in the seminiferous tubules increased significantly with age, and the cells moved closer to the basement region of the seminiferous tubules (**Figures 1C, D**).

### Overview of the Sequencing of lncRNA and mRNA in Cattle Testes

To investigate the roles of mRNAs and lncRNAs in testis development, six cDNA libraries for neonatal (N1, N2, N3) and mature (M1, M2, M3) were constructed. Raw reads were obtained after sequencing by the Illumina HiSeq 4000 Platform. The numbers of raw reads per sample were between 94,866,620 and 115,633,006. 91,525,762 to 112,007,198 clean reads for each sample were obtained, and approximately 14 Gb data per sample was remained by removing low-quality sequences and adaptor sequences (**Table S1**). The GC contents were 46.39–55.24%. More than 94.17% clean reads were mapped to the *B. taurus* UMD3.1, and 389,236 assembled transcripts were produced.

**Figure 2:**
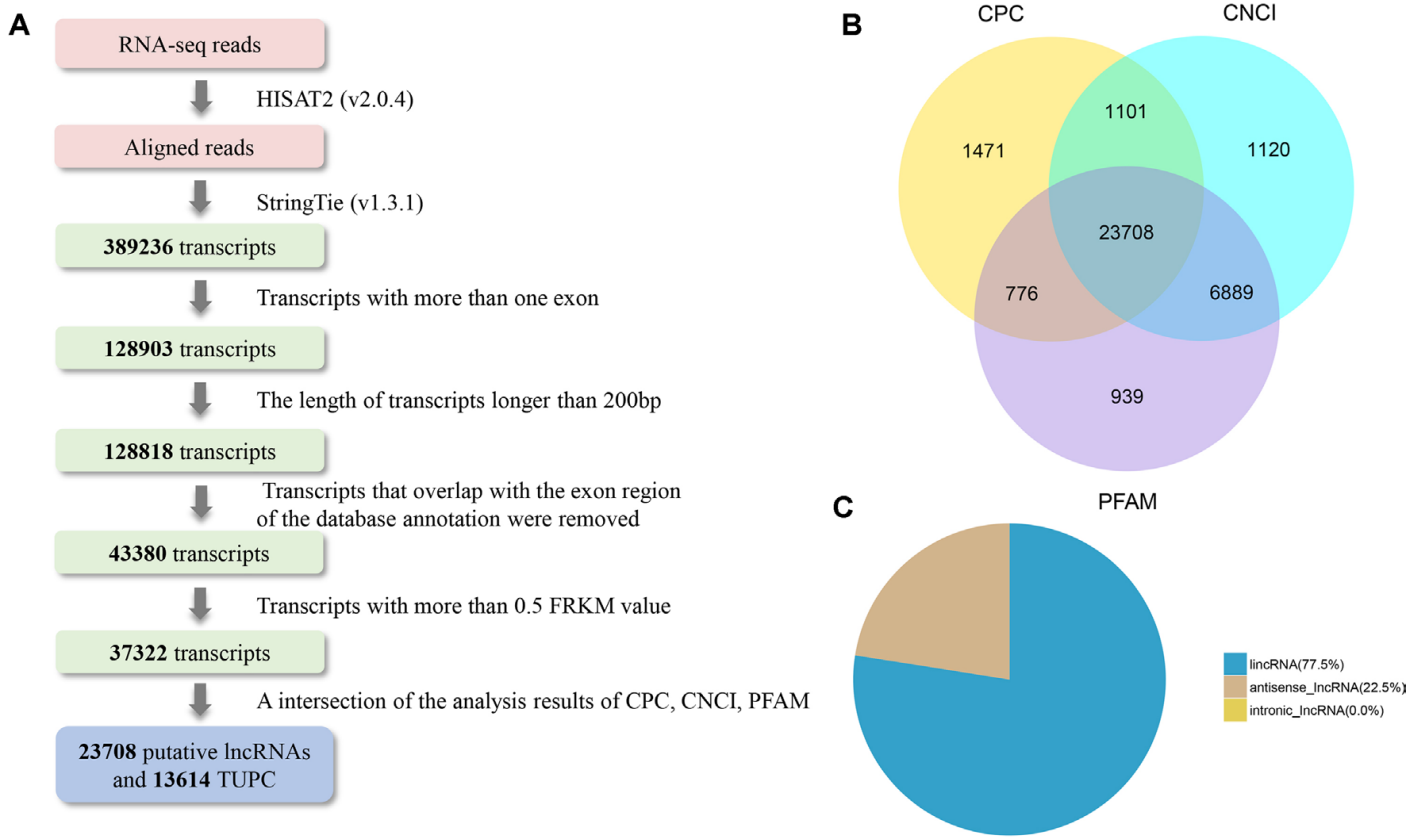
LncRNA identification of bovine testis in postnatal development. **(A)** Integrative pipeline for the identification of putative lncRNAs in this study. **(B)** Screening lncRNAs in bovine testes by an intersection of the result of CPC, phyloCSF, and CNCI analyses. By using the three tools to analyze the protein-coding lncRNAs, 23,708 lncRNAs were identified after the removal of putative protein-coding transcripts. **(C)** Distribution of the three types of lncRNAs.

On the basis of the structural features and non-coding functional characteristics of lncRNAs, We identified reliable putative lncRNAs from assembled transcripts with a stringent pipeline (**Figure 2A**). Finally, a total of 23,708 putative lncRNAs were identified (**Figure 2B**), which included 77.5% lincRNAs and 22.5% antisense lncRNAs (**Figure 2C**). In addition, 27 annotated lncRNAs were detected in the Ensemble database. To avoid filtering out potential lncRNAs, 13,614 TUCP were separated. Moreover, 22,118 protein-coding transcripts were found at the two development stage libraries. From the above, 23,735 lncRNAs (27 annotated and 23,708 candidate lncRNAs), 13,614 TUCP, and 22,118 protein-coding transcripts were screened for further analysis. **Tables S2**, **S3**, and **S4** listed the information of identified lncRNAs, protein-coding transcripts, and TUPC.

### Genomic Characteristics and Expression Profiling of lncRNAs and mRNAs

**Figure 3:**
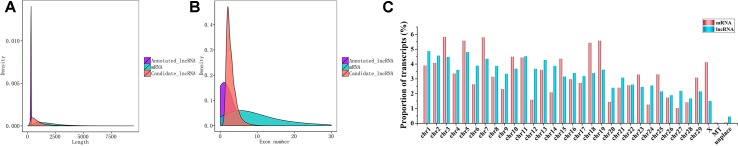
Seqauence features of lncRNAs. **(A)** Length distribution of 27 annotated (purple) and 23,708 candidate lncRNAs (red) and 22,118 mRNAs (blue). **(B)** Exon number distribution of annotated and candidate lncRNAs and mRNA. **(C)** Distribution of testis lncRNA and mRNA on the bovine chromosome.

In the study, we found lncRNAs (1,210 bp on average) were significantly shorter than the mRNAs (2,040 bp on average) (**Figure 3A**). The number of exons of mRNAs (10.03 on average) was more than that of lncRNAs (2.92 on average). Seventy-seven point thirty-six percent of lncRNAs have three or fewer exons, while 69.38% of mRNAs have five or more exons (**Figure 3B**). In addition, we found that there was none lncRNA located in the mitochondria (**Figure 3C**).

**Figure 4:**
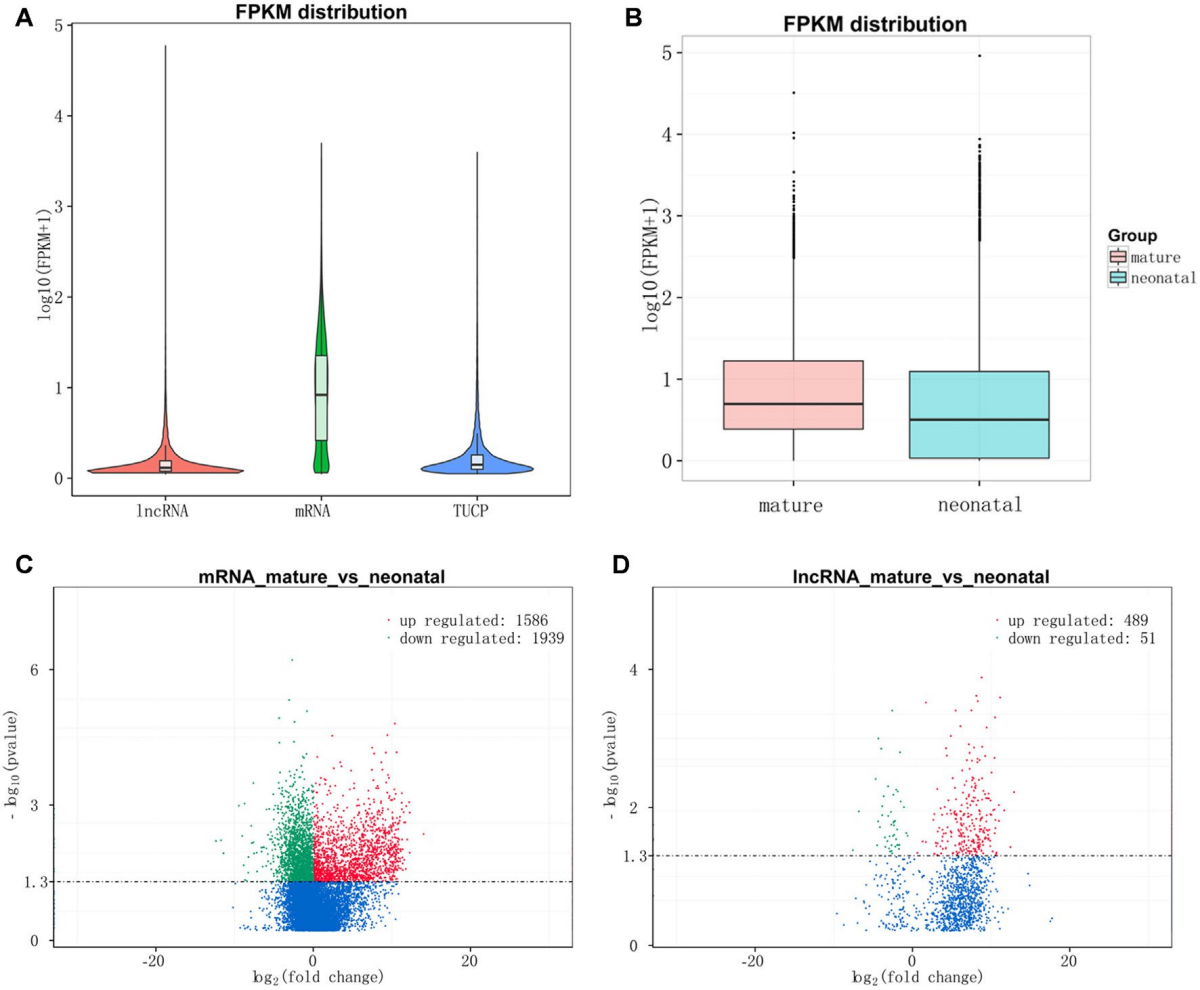
Expression levels of lncRNAs and protein-coding genes. **(A)** The expression level of lncRNAs, mRNAs, and TUPC based on log10(FPKM+1). **(B)** Box plots of lncRNAs, protein-coding genes, and TUPC expression level (log 10 FPKM+1) of neonatal and mature testes group. **(C)** Differential mRNA expression between neonatal and mature bovine testis. **(D)** Differential lncRNA expression between neonatal and mature bovine testis. Volcano plots showed –log10 (*P* value) versus log2 (fold change) difference in mRNA and lncRNA abundance in FPKMs between neonatal and mature cattle testis. Red circles denoted significantly up-regulated lncRNAs, whereas green circles denoted significantly downregulated lncRNAs (*P* < 0.05).

After quantifying the expression level by Cuffdiff and Ballgown, lncRNAs and TUPC exhibited a lower expression level than mRNAs (**Figure 4A**) and the overall expression level of neonatal testes group was lower than mature testes group (**Figure 4B**). Then, we detected the differentially expressed lncRNAs and mRNAs between neonatal and mature testes by using Ballgown. As a result, 540 lncRNAs and 3,525 mRNAs were significantly differentially expressed (DE) between immature and mature bovine testes. Furthermore, we identified 489 lncRNAs significantly upregulated and 51 lncRNAs downregulated in the adult testes group (*P*-value < 0.05). Taking *P*-adjust < 0.05 as the cutoff, 1,586 mRNAs were found to be up-regulated and 1,939 mRNAs were down-regulated in the adult testes group. The volcano plot displayed differentially expressed lncRNA (**Figure 4C**) and mRNAs (**Figure 4D**). **Tables S5** and **S6** listed the differentially expressed lncRNAs and mRNAs, respectively.

### Enrichment Analysis of DE mRNAs

**Figure 5:**
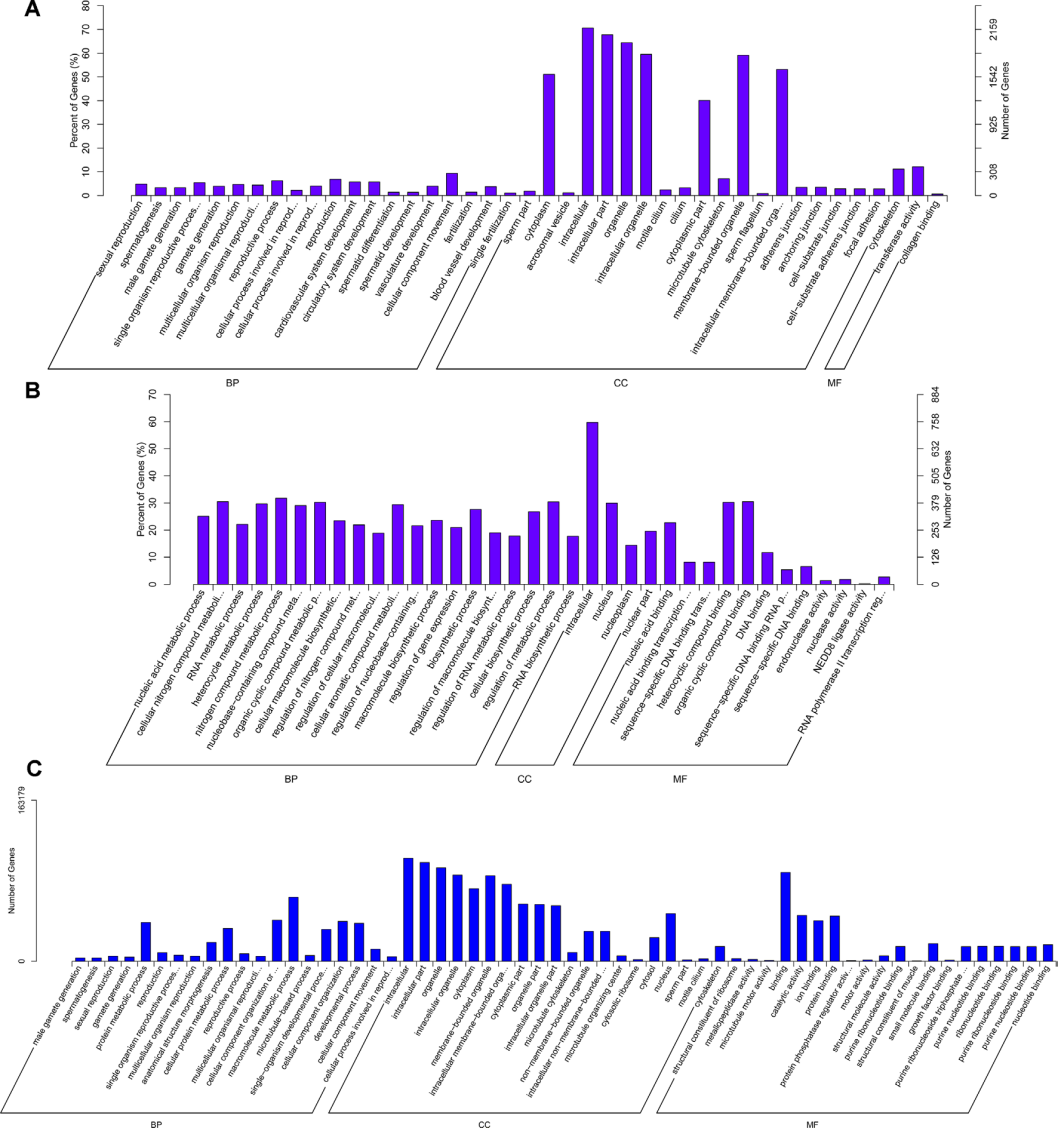
GO enrichment results of mRNA **(A)**, cis-regulated target genes of lncRNAs **(B)**, and trans-regulated target genes of lncRNAs **(C)**. BP, biological process; MF, molecular function; CC, cellular component. The left and right *y*-axes represent the percentage and number of genes, respectively.

**Figure 6:**
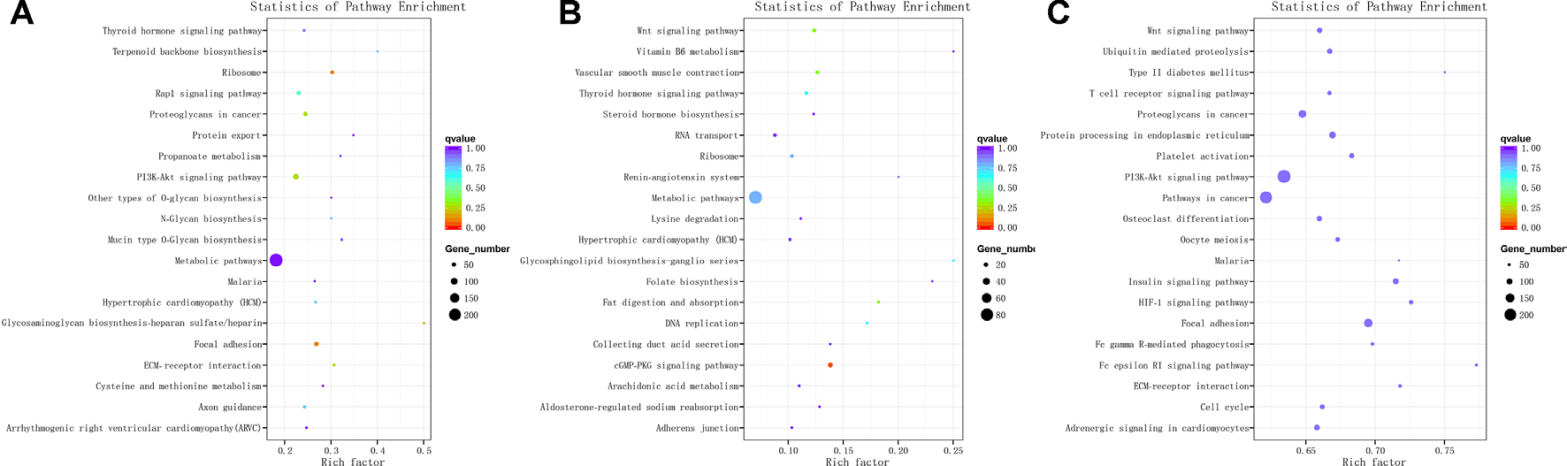
KEGG enrichment for mRNA **(A)**, cis-regulated target genes of lncRNAs **(B)**, and trans-regulated target genes of lncRNAs **(C)**. The top 20 significant enriched KEGG pathways are listed for protein-coding genes **(A)** and cis-regulated **(B)** and trans-regulated **(C)** target genes of lncRNAs, respectively.

The 3,084 DE mRNAs were annotated, and these mRNAs were selected for GO enrichment analysis. The DE mRNAs were significantly enriched to GO terms, including sperm part, sexual reproduction, spermatogenesis, male gamete generation, and single organism reproductive process, which were top five GO terms (**Figure 5A**). As for the pathways, DE mRNAs were enriched in several KEGG pathways such as the focal adhesion pathway and PI3K–Akt signaling pathway (**Figure 6A**). In total, 241 DE genes (158 up-regulated and 83 down-regulated) were enriched in GO terms related to male reproduction concerning spermatogenesis and sperm part (**Table S7**). The upregulated genes *FSCN3*, *TNP2*, *IQCF1*, *PRM2*, and *LOC516410* and downregulated genes *MORC1*, *TMEM119*, *MERTK*, *GGT1*, and *ADAMTS2* were enriched for spermatogenesis. Moreover, the upregulated genes *KLHL10*, *CCDC182*, and *SPINK2B* and the downregulated genes *TCF21*, *SLIT2*, and *SFRP1* were enriched in male gonad development. Additionally, the downregulated genes *AR*, *SFRP1*, and *TCF21* and the upregulated genes *KDM3A* and *DNAJA1* were enriched in the androgen receptor signaling pathway, which might affect androgen synthesis. Moreover, 233 DE genes (55 up-regulated and 178 down-regulated) were enriched in 17 KEGG pathways related to male reproduction (**Table S8**). For example, *RELN*, *COMP*, and *COL4A6* were enriched for the PI3K–Akt pathway. In order to investigate the key genes related to bovine sexually maturation, 138 DE genes (FPKM values > 10 and |fold change| > 4) were chosen for further analysis (**Table S9**).

### LncRNAs Target Genes of *Cis*- or *Trans*-Regulated

We predicted the targets of lncRNAs for elucidating lncRNAs function. Taking 100 kb as the cutoff, 1,166 mRNAs were found as nearest neighbors of 411 out of 540 lncRNAs. GO enrichment analysis results showed that 70 significantly GO terms were observed (corrected *P*-value < 0.05). The top five GO terms were nucleic acid binding, nucleic acid metabolic process, nucleic acid binding transcription factor activity, sequence-specific DNA binding transcription factor activity, and cellular nitrogen compound metabolic process (**Figure 5B**). KEGG analysis identified 12 pathways (*P* < 0.05), and the top 20 pathways were shown in **Figure 6B**, such as Wnt signaling pathway, steroid hormone biosynthesis, adherens junction, and cyclic guanosine 3’, 5’-monophosphate (cGMP)-dependent protein kinase (PKG) signaling pathway.

We also investigated the target genes of lncRNAs in a trans manner. Using the Pearson correlation ≥ 0.95 as the cutoff, 540 lncRNAs and 11,470 genes were detected. GO enrichment analysis results showed that 193 significantly GO terms were identified and the top five GO terms were spermatogenesis, male gamete generation, sexual reproduction, gamete generation, and reproduction (**Figure 5C**). Concerning the pathways, the *trans*-target genes for lncRNAs were enriched in several KEGG pathways, for example, the Wnt signaling pathway, PI3K–Akt signaling pathway, oocyte meiosis, insulin signaling pathway, and focal adhesion (**Figure 6C**).

**Figure 7:**
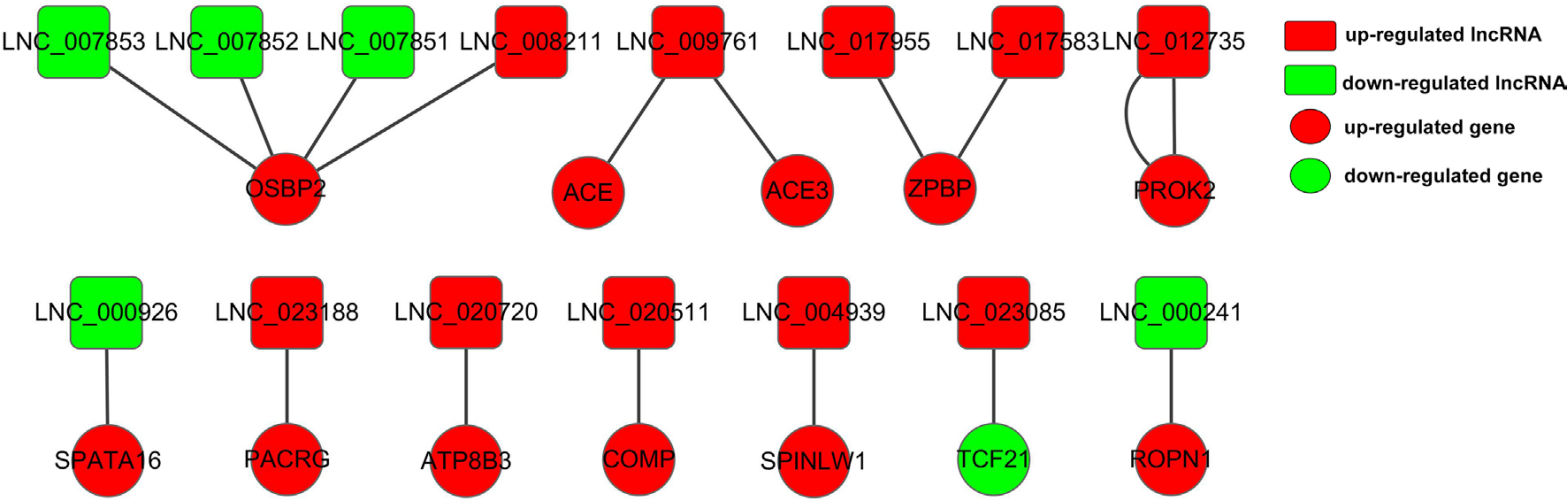
Constructed network of the interaction between 15 DE lncRNAs and 12 cis-targets. The red and green color represent up- and down-regulation, and the box and roundness represent DE lncRNAs and genes, respectively.

Moreover, we chose 138 DE genes related to male reproduction to construct lncRNA–gene interaction network involving 15 DE lncRNAs and 12 *cis*-targets (**Table S10**, **Figure 7**), as well as 413 DE lncRNAs and 138 *trans*-targets (**Table S11**). LNC_023188, LNC_020720, and LNC_020511 played *cis* roles to regulated *PACRG*, *ATP8B3*, and *COMP*, respectively. The upregulated DE gene *OSBP2* was regulated by three downregulated DE lncRNAs (LNC_007851, LNC_007852, and LNC_007853) and one upregulated DE lncRNA (LNC_008211). *ZPBP* interacted with two lncRNAs, LNC_017583 and LNC_017955. Meanwhile, LNC_009761 was targeting-regulated by *ACE* and *ACE3*. Moreover, the upregulated lncRNA LNC_023085 was targeting-regulated by the downregulated gene *TCF21*, and two genes (*SPATA16* and *ROPN1*) that are highly expressed in adult bovine testis interacted downregulated lncRNAs LNC_000926 and LNC_000241.

### Verification of DE lncRNA and mRNA Profiling

**Figure 8:**
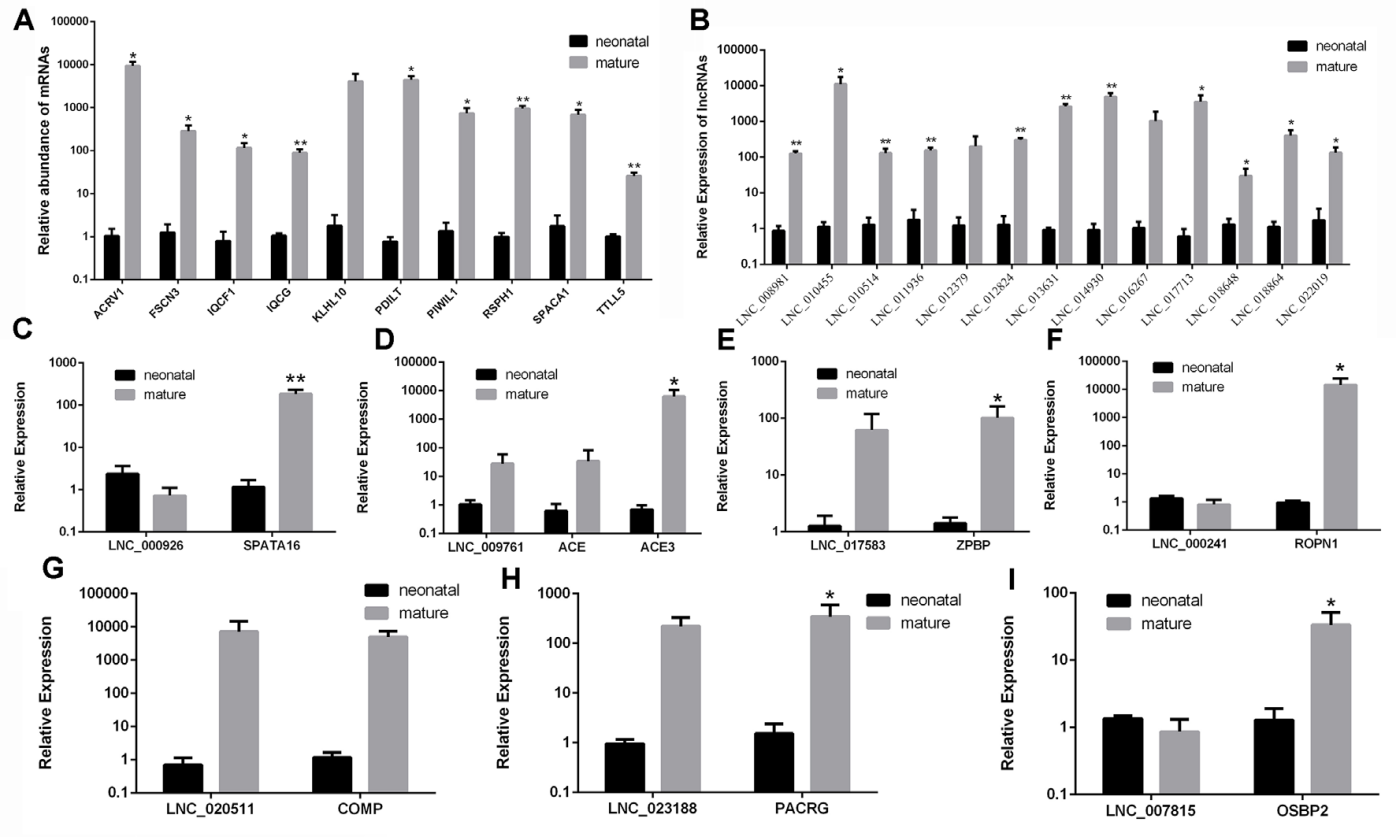
Validation of differentially expressed mRNAs and lncRNAs in two development stages by RT-qPCR. **(A)** RT-qPCR validation of mRNA expression changes between neonatal and mature cattle testis. **(B)** RT-qPCR validation of lncRNA expression changes between neonatal and mature cattle testis. Panels **(C–I)** show the expression levels of seven DE lncRNAs (LNC_000926, LNC_009761, LNC_017583, LNC_000241, LNC_020511, LNC_023188, and LNC_007815) and their target genes (SPATA16, ACE, ACE3, ZPBP, ROPN1, COMP, PACRG, and OSBP2) in two testes stages. The experiment was biologically repeated three times and technically repeated three times for each group. RT-qPCR data were calculated by the 2−ΔΔCt method with β-actin as an internal control. The data are present as the mean ± standard deviation of the mean; **P < 0.01, *P < 0.05. The *Y*-axis represents the values that were calculated by log10.

For the purpose of validating the RNA-seq results, we randomly selected differentially expressed 10 mRNAs and 13 lncRNAs using RT-qPCR (**Table S12**). The primer details are presented in **Table S13**. The relative expression in RT-qPCR corresponded to RNA-seq results (**Figures 8A, B**). In conclusion, the results showed that the RNA-seq data are accurate and reliable with the high specificity of the developmental stage.

To further validate whether the identified DE lncRNAs play roles in male reproduction, seven candidate lncRNAs, LNC_000926, LNC_009761, LNC_017583, LNC_000241, LNC_020511, LNC_023188, and LNC_007815, along with their cis-target genes, *SPATA16*, *ACE*, *ACE3 ZPBP*, *ROPN1*, *COMP*, *PACRG*, and *OSBP2*, were screened and examined during testis development. The expression of these lncRNAs and their target genes was examined in the testes in the neonatal and mature groups (**Figures 8C–I**). As shown in **Figures 8D, E, G, H**, the expression patterns of LNC_009761, LNC_017583, LNC_020511, and LNC_023188 were similar to those of their target genes during testis development. Instead, the expression patterns of LNC_000926, LNC_000241, and LNC_007815 were opposite to those of their target genes.

### Expression of mRNA and lncRNA in Different Cells of Testis

**Figure 9:**
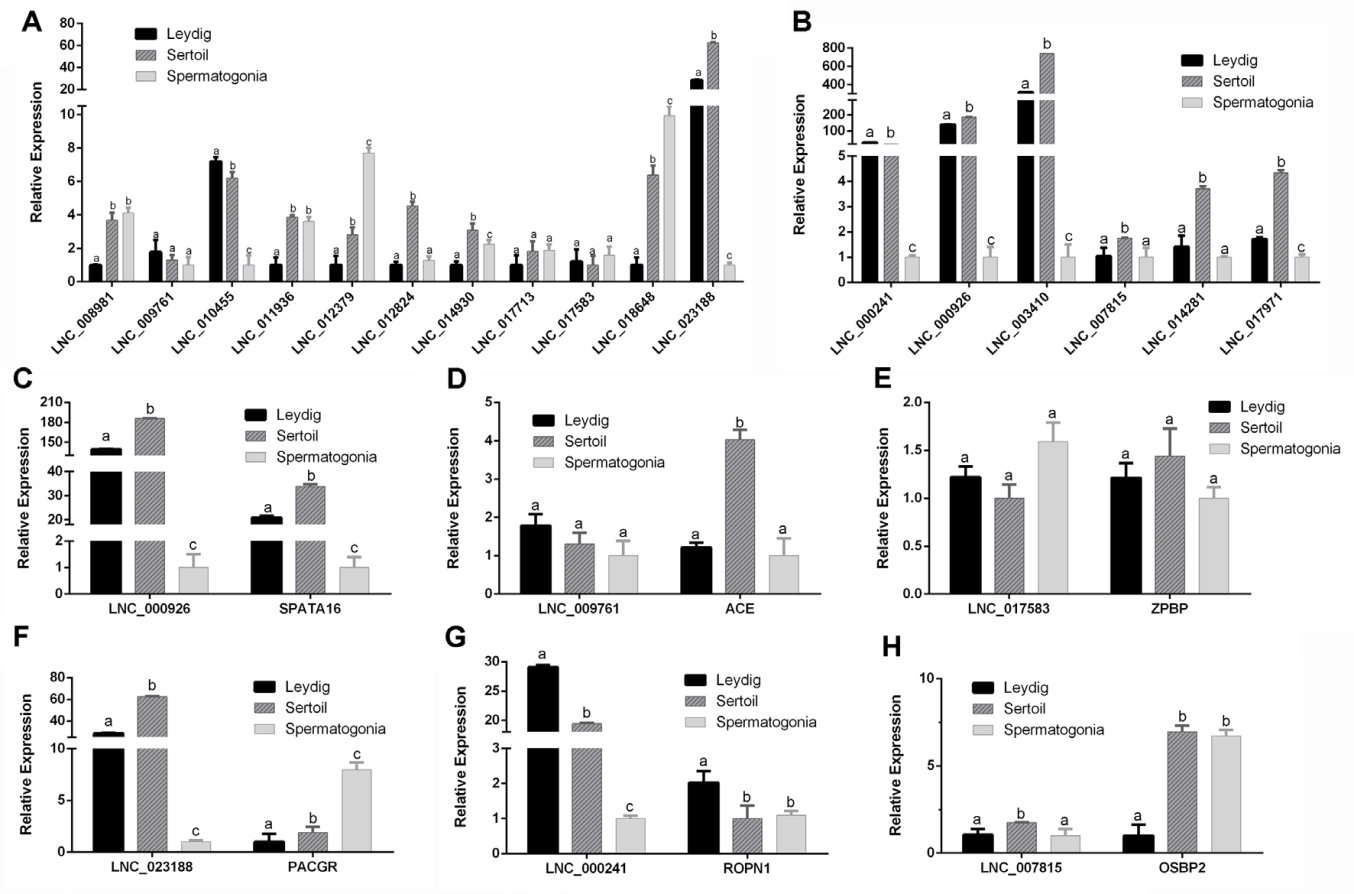
The expression levels of lncRNAs and cis-target genes at Leydig cells, Sertoli cells, and spermatogonia. **(A)** The expression levels of up-regulated lncRNAs at Leydig cells, Sertoli cells, and spermatogonia. **(B)** The expression levels of down-regulated lncRNAs at Leydig cells, Sertoli cells, and spermatogonia. Panels **(C–H)** show the expression levels of six DE lncRNAs and their target genes at Leydig cells, Sertoli cells, and spermatogonia. RT-qPCR data were calculated by the 2−ΔΔCt method with β-actin as an internal control. The data are present as the mean ± standard deviation of the mean. a, b, c: different letters denote statistically significant differences within each group.

To further validate the roles of lncRNA in male reproduction, the different cell types were isolated and the expression of mRNA and lncRNA in bovine Leydig cells, Sertoli cells, and spermatogonia was detected. The marker genes of the different cell were examined (**Figure S1**, **Table S13**). Eleven up-regulated lncRNAs and six down-regulated lncRNAs were chosen to analyze their expression (**Figures 9A, B**). As shown in **Figure 9A**, five lncRNAs (LNC_008981, LNC_012379, LNC_017713, LNC_017583, and LNC_018648) had the highest expression in spermatogonia. LNC_008981, LNC_012379, LNC_017713, and LNC_018648 were low expressed in Leydig cells, and LNC_017583 has low expression in Sertoli cells. Besides, LNC_009761 and LNC_010455 were highly expressed in Leydig cells. Furthermore, LNC_011936, LNC_012824, LNC_014930, and LNC_023188 were highly expressed in Sertoli cells. The results showed that all detected down-regulated lncRNAs have low expression in spermatogonia. Five lncRNAs (LNC_000926, LNC_003410, LNC_007815, LNC_014281, and LNC_017971) were highly expressed in Sertoli cells, and LNC_000241 had the highest expression in Leydig cells.

In order to better determine the relationship between lncRNA and target genes, the expression of their target genes was detected in different cells. The corresponding expression of lncRNAs and their target genes was shown in **Figures 9C–H**. Interestingly, LNC_000926, LNC_000241, and LNC_007815 along with their target genes *SPATA16*, *ROPN1*, and *OSBP2* had opposite expression trend during testis development but had consistent expression trend in different cells. These results revealed that these lncRNAs might play important roles during testis development.

## Discussion

Testis development and spermatogenesis are complex biological processes that are controlled by well-coordinated gene regulation, including coding genes and non-coding RNAs (Card et al., 2013; Djureinovic et al., 2014). Testis development includes the formation of testis tissue during embryonic and the growth of testis tissue at the postnatal stage (Svingen and Koopman, 2013). The testes of the bull grow relatively slowly until approximately 25 weeks of age and then a rapid phase of growth occurs until puberty, at 37–50 weeks of age (Rawlings et al., 2008). In order to explore the process of sexual maturation in the bull calf, 3 days after birth (neonatal, pre-sex maturation) and 13 months old (mature, post-sex maturation) (Lunstra, 1982), Angus bulls were selected. Long non-coding RNAs (lncRNAs) are a class of transcribed RNA molecules with a length of more than 200 nucleotides that do not encode proteins. However, lncRNAs as key regulators in multitudinous biological processes have not been systematically identified in bovine testes during sexual maturation. In this study, we identified and characterized lncRNAs in bovine testes and explored potential genes and lncRNAs that regulated testis development.

A total of 23,735 lncRNAs (27 annotated and 23,708 candidate lncRNAs), 13,614 TUCP, and 22,118 protein-coding transcripts were identified in bovine testes at two development stages. Zhu et al. (2016) reported that the number of lncRNAs in testis is the highest of all organs. Evidence suggested that the human lincRNAs exhibited more tissue-specific mRNAs and one-third of the 8,000 human lincRNAs are specifically expressed in the testis (Cabili et al., 2011). The number of lncRNAs in our study was significantly higher than those reported from skin (4,848 lncRNAs) (Weikard et al., 2013), oocytes and early embryos (11,220 lncRNA) (Caballero et al., 2014), 18 cow tissues (16,336 lncRNA) (Koufariotis et al., 2015), skeletal muscle (7,692 lncRNAs) (Sun et al., 2016), and mammary glands (184 lincRNAs) (Tong et al., 2017), revealing that lncRNAs are testis-specific.

Fewer exons were tested within 23,735 identified lncRNAs, and shorter lengths as well as lower expression levels than mRNAs (**Figure 3**). We found that this comparison analysis of genomic characteristic between lncRNAs and mRNAs is in accordance with recent studies in other mammals (Ran et al., 2016; Wen et al., 2016; Liu et al., 2017; Zhang et al., 2017). These general features of lncRNAs may indicate that mammals have some conservative regulation. Furthermore, 540 lncRNAs and 3,525 mRNAs were differentially expressed between two stages. The result of RT-qPCR confirmed that lncRNA and mRNA expression has a high correlation with the transcriptome data (**Figure 5**). Previous studies demonstrated that lncRNAs exhibited high developmental stage-specificity in mouse and pig testes (Sun et al., 2013; Ran et al., 2016). Our analyses also suggested that the lncRNAs found here were specifically expressed in the differential developmental stage in bovine testis.

To deeply explore the biological functions of lncRNAs and mRNA in bovine testis, GO and KEGG analyses were performed for target genes of differentially expressed lncRNAs and DE mRNAs. Through these analyses, we found various candidate genes and some of these genes have been demonstrated to be related to male reproduction. *FSCN3*, *TNP2*, *IQCF1*, *PRM2*, and* MORC1* were related to spermatogenesis. *KLHL10*, *TCF21*, and *SFRP1* may play roles in male gonad development and the androgen receptor. *RELN*, *COMP*, and *COL4A6* were enriched for PI3K–Akt pathway, which played a functional role in both Sertoli cells (Sun et al., 2015) and Leydig cells (Zhuo et al., 2016; Sun et al., 2017).

Most evidence suggests that the expression of lncRNAs can regulate and have high correlations with an expression of neighboring mRNAs (Ponting et al., 2009). In this study, the *cis*-target genes of 540 differentially expressed lncRNAs were used to predict their potential roles in the regulation of testis development and spermatogenesis. Combining with 138 DE male reproduction-related genes, construct lncRNA–gene interaction network involving 15 DE lncRNAs and 12 *cis*-targets show their potential in male reproduction regulation. LNC_000926 was predicted to be regulated by the target gene *SPATA16*, one gene of SPATA (spermatogenesis associated) gene family that plays a vital role in testis development and spermatogenesis (Wu et al., 2010). The *SPATA16* protein localizes to the Golgi apparatus and the proacrosomal granules that are transported in the acrosome in the round and elongated spermatids (Xu et al., 2003). Mutations or deletions in *SPATA16* have been shown to be responsible for globozoospermia (De Braekeleer et al., 2015). Dam et al. (2007) identified a homozygous mutation (c.848G.A) in exon 4 of the *SPATA16* gene in three brothers affected with globozoospermia from a consanguineous Ashkenazi Jewish family. This mutation leads to the substitution of an arginine to a glutamine at residue 283 (R283Q) in the protein (Dam et al., 2007). Moreover, *SPATA1*, *SPATA9*, *SPATA16*, *SPATA6*, *SPATA3*, *SPATA20*, *SPATA18*, *SPATA25*, and *SPATA46* were up-regulated in the mature testis.

*RONP1* is predicted to be a target gene of LNC_00024 and is differentially expressed in two development stages. *ROPN1* (ropporin 1), a protein kinase A-like (R2D2) protein, is associated with cAMP dependent protein kinase (PKA)/A-kinase anchoring protein (AKAP). Fujita et al. reported that *ROPN1* is localized to the sperm flagella (Fujita et al., 2000). Another study reported decreased expression of *ROPN1* in the spermatozoa of patients with low sperm motility (Chen et al., 2011). Furthermore, Fiedler et al. demonstrated that *ROPN1* is essential for murine sperm motility, phosphorylation, and fibrous sheath integrity (Fiedler et al., 2013). Suppression of SUMOylation of *ROPN1* and *ROPN1L* partially mediates the effects of *FSCB* phosphorylation on the mobility of mouse spermatozoa (Xinqi Zhang, 2016).

*OSBP2* was targeting-regulated by four DE lncRNAs (LNC_007851, LNC_007852, LNC_007853, and LNC_008211). LNC_007851 and *OSBP2* were verified in testis development stages and different cells. The result showed that LNC_007851 and *OSBP2* have an opposite expression in two development stages but have a consistent expression trend in different cells. *OSBP2* (oxysterol-binding protein 2) is detected mainly in retina, testis, and fetal liver (Moreira et al., 2001). *OSBP2* has been reported to be essential for the post-meiotic differentiation of germ cells, while the lack of *OSBP2* (*ORP4*) caused male infertility owing to oligo-astheno-teratozoospermia (Charman et al., 2014; Udagawa et al., 2014). This may imply that *OSBP2* is significantly associated with bovine spermatogenesis.

Angiotensin-converting enzyme (ACE), a carboxypeptidase that removes C-terminal dipeptides from substrates, was the putative target gene of LNC_009761. The activity of testis-specific isoform of angiotensin-converting enzyme (tACE) may be required for bovine sperm capacitation (Costa and Thundathil, 2012; Ojaghi et al., 2017). A predicted target of LNC_023085 is the basic helix-loop-helix (bHLH) gene *TCF21*, which was one of the direct downstream targets of *SRY*. *SRY* directly acts on the *TCF21* promoter to regulate Sertoli cell differentiation and embryonic testis development (Bhandari et al., 2011; Bhandari et al., 2012).

In conclusion, this study provided the first comprehensive analysis of the mRNA and lncRNA expression profiles in immature and mature bovine testes. Also, the list of lncRNAs generated from our study was a valuable resource on understanding their regulatory roles in bovine testes development and spermatogenesis. In addition, the useful insights could be provided in further bovine reproductive performance regulation researches involving these lncRNAs and genes.

## Data Availability

The sequencing data have been deposited in the Sequence Read Archive (SRA) database (accession number: SRP148084).

## Ethics Statement

All experimental design and procedures were performed by the Regulations for the Administration of Affairs Concerning Experimental Animals (Ministry of Science and Technology, China, 2004). The study was approved by the Institutional Animal Care and Use Committee of Northwest A&F University.

## Author Contributions

RD designed and conceptualized the experiments. YG, ZL, and SL performed the experiments. ZZ, FW, and YH analyzed the data. XL, CL, and HC participated in collecting animals testes. YG drafted the manuscript. All authors read and approved the final manuscript.

## Funding

This work is financially supported by the Key Research Development Plan of Shaanxi Province of China (General Project) (2017NY-071), the Science & Technology Project of Yangling of China (2018NY-33), Program of the National Beef Cattle and Yak Industrial Technology System (CARS-37), and National Natural Science Foundation of China (81770514).

## Conflict of Interest Statement

The authors declare that the research was conducted in the absence of any commercial or financial relationships that could be construed as a potential conflict of interest.

## Abbreviations

CNCI, Coding-Non-Coding-Index; CPC, coded potential calculator; DAVID, database for annotation, visualization, and integrated discovery; FPKM, fragments per kilobase of transcript per million mapped reads; WGCNA, weighted gene co-expression network analysis; GO, Gene Ontology; KEGG, Kyoto Encyclopedia of Genes and Genomes; lncRNAs, long non-coding RNAs; TUCP, transcripts of uncertain coding potential; RT-qPCR, real-time quantitative polymerase chain reaction; NCBI, National Center for Biotechnology Information; SRA, Sequence Read Archive.
