# Supplementary material for: Analysis of Long Non-Coding RNA and mRNA Expression Profiling in Immature and Mature Bovine (Bos taurus) Testes
Source: Front Genet. 2019 Jul 5;10:646. doi: 10.3389/fgene.2019.00646 (PMC6624472; doi:10.3389/fgene.2019.00646)
Supplement: Supplementary file 1 [file Table_1.docx]

**Table S1. Summary of reads and matches.**

| Sample name | M1 | M2 | M3 | N1 | N2 | N3 |
| --- | --- | --- | --- | --- | --- | --- |
| Raw reads | 94866620 | 99355310 | 98676988 | 97715528 | 96661530 | 115633006 |
| Clean reads | 91525762 | 96639934 | 95105056 | 94414154 | 93742744 | 112007198 |
| Clean bases | 13.73G | 14.5G | 14.27G | 14.16G | 14.06G | 16.8G |
| GC content(%) | 46.39 | 49.37 | 48.17 | 51.84 | 52.2 | 51.03 |
| Total mapped | 86955668 (95.01%) | 91991361 (95.19%) | 89562538 (94.17%) | 89337136 (94.62%) | 89229051 (95.19%) | 106066039 (94.7%) |
