# Supplementary material for: Analysis of Long Non-Coding RNA and mRNA Expression Profiling in Immature and Mature Bovine (Bos taurus) Testes
Source: Front Genet. 2019 Jul 5;10:646. doi: 10.3389/fgene.2019.00646 (PMC6624472; doi:10.3389/fgene.2019.00646)
Supplement: Supplementary file 12 [file Table_12.docx]

Table S12. Expression patterns of RNA-seq for the 23 selected genes as verified by RT-qPCR.

| Transcript ID | Gene ID | Gene name | Log_2_ (fold-change) | *p*-Value |
| --- | --- | --- | --- | --- |
| ENSBTAT00000047491 | ENSBTAG00000033384 | *ACRV1* | 11.2392252 | 0.0005444 |
| ENSBTAT00000065372 | ENSBTAG00000009365 | *FSCN3* | 11.35677447 | 0.0198382 |
| ENSBTAT00000001065 | ENSBTAG00000000805 | *IQCF1* | 10.73373465 | 0.0114379 |
| ENSBTAT00000023995 | ENSBTAG00000018025 | *IQCG* | 4.036987216 | 0.0333497 |
| ENSBTAT00000036408 | ENSBTAG00000011459 | *KLHL10* | 10.06527652 | 0.0066271 |
| ENSBTAT00000021853 | ENSBTAG00000016434 | *PDILT* | 9.712299591 | 0.011357 |
| ENSBTAT00000008482 | ENSBTAG00000006468 | *PIWIL1* | 7.051560708 | 0.0061909 |
| ENSBTAT00000043552 | ENSBTAG00000021284 | *RSPH1* | 8.570611502 | 0.0083758 |
| ENSBTAT00000025934 | ENSBTAG00000019469 | *SPACA1* | 5.842603731 | 0.0035656 |
| ENSBTAT00000035678 | ENSBTAG00000025403 | *TTLL5* | 4.101860943 | 0.0086997 |
| LNC_008981 | XLOC_105286 | - | 9.980101256 | 0.0127593 |
| LNC_010455 | XLOC_120596 | - | 9.683052917 | 0.0073784 |
| LNC_010514 | XLOC_121527 | - | 8.322119732 | 0.0294104 |
| LNC_011936 | XLOC_139402 | - | 7.914437374 | 0.0044389 |
| LNC_012379 | XLOC_144938 | - | 10.16219884 | 0.0432962 |
| LNC_012824 | XLOC_150031 | - | 6.396838292 | 0.0060968 |
| LNC_013631 | XLOC_158443 | - | 6.982569097 | 0.0100854 |
| LNC_014930 | XLOC_171814 | - | 12.87912678 | 0.0059691 |
| LNC_016267 | XLOC_187483 | - | 6.391085273 | 0.0234504 |
| LNC_017713 | XLOC_205810 | - | 10.51399372 | 0.0199325 |
| LNC_018648 | XLOC_219154 | - | 7.52979378 | 0.0020962 |
| LNC_018864 | XLOC_221826 | - | 5.913192497 | 0.0221516 |
| LNC_022019 | XLOC_263688 | - | 8.234367429 | 0.0022075 |
