# Supplementary material for: Analysis of Long Non-Coding RNA and mRNA Expression Profiling in Immature and Mature Bovine (Bos taurus) Testes
Source: Front Genet. 2019 Jul 5;10:646. doi: 10.3389/fgene.2019.00646 (PMC6624472; doi:10.3389/fgene.2019.00646)
Supplement: Supplementary file 13 [file Table_13.docx]

Table S13. List of primers used in the RT-qPCR at two development stages. Tm=60℃.

| LncRNA ID | Primer | Product length (nt) |
| --- | --- | --- |
| LNC_008981 | F: 5′-CGGAGGGTCTGTAGTGGTC-3′  R: 5′-GGGATGGAGACAGGGAGTG-3′ | 189 |
| LNC_010455 | F: 5′-CACAGACTGAACCCGTCACT-3′  R: 5′-GATCAAAGCCCCAAGACATGC-3′ | 137 |
| LNC_010514 | F: 5′-CACTTCCTTGGCAGGTCTT-3′  R: 5′-AACCCAGGTCTCCAGCATT-3′ | 198 |
| LNC_011936 | F: 5′-GGACACATTGCTCTGTGGTTT-3′  R: 5′-GGCACGTGGACTGAGCTATC-3′ | 199 |
| LNC_012379 | F: 5′-GCCATACCTTCTAAGCGTTCT-3′  R: 5′-TTCCTCCTCTTCCAGTCGTT-3′ | 173 |
| LNC_012824 | F: 5′-GAGCTTTCCAAACAGCAGCC-3′  R: 5′-AGGTTCAAGTCCCCAAAAGACA-3′ | 246 |
| LNC_013631 | F: 5′-TTAAAGGGGCTGTTTGGGAGG-3′  R: 5′-AGCAGCATAGAGAAGGGTGG-3′ | 248 |
| LNC_014930 | F: 5′-CAAGAAGCAGCAACTACCG-3′  R: 5′-CAAGAAGCAGCAACTACCG-3′ | 160 |
| LNC_016267 | F: 5′-GTGCCTGGAACATTGCTTAG-3′  R: 5′-CTGTGGGATCTTTAGTTATGG-3′ | 183 |
| LNC_017713 | F: 5′-CAGCAAGGCAGCACCATCT-3′  R: 5′-CAGGCAATAACGATCTTTGTGAA-3′ | 123 |
| LNC_018648 | F: 5′-CAGGTCTGCATTCAAGGGGG-3′  R: 5′-GGCAGACACTTTCCTGGCATA-3′ | 248 |
| LNC_018864 | F: 5′-TCCAGGTGTAGGTAGTCCCTG-3′  R: 5′-GCATCCCTGGATAGGCTCAA-3′ | 213 |
| LNC_022019 | F: 5′-GCTGTTTCTCAAGTGCTGGC-3′  R: 5′-ACTCCTAGGGGATGGCCTTT-3′ | 245 |
| LNC_000926 | F: 5′- TCTCCTTTCACGCGCTTCTT -3′  R: 5′- ATGGGAGGCAACTTCTGACG -3′ | 124 |
| LNC_009761 | F: 5′- GTCCCTGTCATGCGTTGGTA -3′  R: 5′- AAAGGCCACCAGGCCTAATC -3′ | 112 |
| LNC_017583 | F: 5′- GGCATGTCCAAGCACCTTTG -3′  R: 5′- GGGTTCTTGTGCTGCCAATG -3′ | 161 |
| LNC_000241 | F: 5′- TTCAGGACAGGAACCCCCAC-3′  R: 5′- AGGCACAAGACAGGTCTGAAT -3′ | 137 |
| LNC_020511 | F: 5′- GCGATAGTGCCATGGGGTAA -3′  R: 5′- AACATCTTCTGCCTGGTCGG -3′ | 119 |
| LNC_023188 | F: 5′- AGAGAATGCCCAGGACCAGT -3′  R: 5′- GATCCAGTGAGGTTGACAGCA-3′ | 164 |
| LNC_007815 | F: 5′- AAAGCCCAATCAGGCGATCT -3′  R: 5′- GAGCAAAGCCTGTGGTAAGC-3′ | 124 |
| *ACRV1* | F: 5′-TCTCAGGCCCACCATTAAGC-3′  R: 5′-ATCCTGGTTCCGTGGGAGAA-3′ | 205 |
| *FSCN3* | F: 5′-TAAGCCCAATGTCGTTGTTCC-3′  R: 5′-CATCACTGCCCTATGGCGTCTCTGG-3′ | 110 |
| *IQCF1* | F: 5′-CAGACCAGCCTAAGACAGTTGAT-3′  R: 5′-GCCTGTATCTTTACTGCTTCTGG-3′ | 235 |
| *IQCG* | F: 5′-TATTGCTCACCTCAAGGACCA-3′  R: 5′-TTCTTCAGTTTTCAGCCGTAG-3′ | 172 |
| *KLHL10* | F: 5′-ATGTGCGTCTCAACACTGCT-3′  R: 5′-TCGTTCCCGTTAAACCCACC-3′ | 145 |
| *PDILT* | F: 5′-GAATTCCAGGCCCTTGGTCA-3′  R: 5′-ACGCCCAATGGCATGTCTAA-3′ | 136 |
| *PIWIL1* | F: 5′-GACGGAGGTCAATTGCAGGA-3′  R: 5′-CCTACGCCATCCCGATACAC-3′ | 196 |
| *RSPH1* | F: 5′-AATGGTGCTCGGTACATCGG-3′  R: 5′-TTTGGTGGGCAAACCACTCT-3′ | 178 |
| *SPACA1* | F: 5′-CCCGAGAATGACAGCGAGACG-3′  R: 5′-TCACCACCAGGGCATCCACTT-3′ | 110 |
| *TTLL5* | F: 5′-AACTGGAGGAGGTGTTGACTT-3′  R: 5′-CTGTTTGGGCTGCTTTATTTT-3′ | 167 |
| *β-actin* | F: 5′-GCGTTACACCCTTTTTCTTGACA-3′  R: 5′-TCACCTTCACCGTTCCAGTTT-3′ | 152 |
| *SPATA16* | F: 5′- ACGTGTTGCCTCAGACAGTT-3′  R: 5′- TGCACGAAAACTCCCCTGTA -3′ | 147 |
| *ACE* | F: 5′- TTCGCAGCCAGCTTCAACTC -3′  R: 5′- CGGGTCGAACAGATCCTTGG -3′ | 177 |
| *ACE3* | F: 5′- GATGTGAAGCGCATGCTGAG -3′  R: 5′- AGGTGGCCATGACTTCTTCG -3′ | 184 |
| *ZPBP* | F: 5′- CACGATGTCCTGAGTGCTGT-3′  R: 5′- TATGACCCAGCAATCGCAGT-3′ | 110 |
| *ROPN1* | F: 5′- AGGATCTCATCCAGTGGGCT-3′  R: 5′- GGACAATCAGTCTGCCACCA-3′ | 165 |
| *COMP* | F: 5′- ACCCAGACCAGCGAAATACG-3′  R: 5′- ATCTGAGTTGGGCACCTTGG -3′ | 185 |
| *PACRG* | F: 5′- CAAGCGCAGGAGCATTCAAA-3′  R: 5′- ACACGTGCTTCCACGCTATT -3′ | 129 |
| *OSBP2* | F: 5′- AACACACCTTGGGATGGCAG -3′  R: 5′- GAGTCCTCCGTGTCGAAGTG -3′ | 243 |
