# Supplementary material for: Analysis of Long Non-Coding RNA and mRNA Expression Profiling in Immature and Mature Bovine (Bos taurus) Testes
Source: Front Genet. 2019 Jul 5;10:646. doi: 10.3389/fgene.2019.00646 (PMC6624472; doi:10.3389/fgene.2019.00646)
Supplement: Supplementary file 14 [file Table_14.docx]

Table S13. List of primers used in the RT-qPCR and the detection of marker genes at Leydig cells, Sertoli cell, and spermatogonia. Tm=60℃.

| LncRNA ID | Primer | Product length (nt) |
| --- | --- | --- |
| LNC_003410 | F: 5′- GATTTTCGGCTCAACGCAGG -3′  R: 5′- TGGCTCCTCTGGTTGTTAGG -3′ | 133 |
| LNC_017797 | F: 5′- TGCGGTAGCGTAGTTTCCTT -3′  R: 5′- ATCCACTTCTGCGTAGCACC -3′ | 167 |
| LNC_014281 | F: 5′- ACTGCTGTAGACCAGGACCA -3′  R: 5′- CCGCATTTAAGGCCTTTCAGA -3′ | 153 |
| PLZF | F: 5′- CACCGCAACAGCCAGCACTAT-3′  R: 5′- CGGCATACAGCAGGTCATCCAA-3′ | 127 |
| DAZL | F: 5′- TCCGTCCTCTGGAAATGGC-3′  R: 5′- AGCACTGCCCGACTTCTTC-3′ | 168 |
| OCT4 | F: 5′- AAGGGCAAACGATCAAGCA-3′  R: 5′- AATGGGACCGAAGAGTACAGAGT-3′ | 167 |
| GFRA1 | F: 5′- CTCCCAGTTCAGACCACCAC -3′  R: 5′- GAGAGGAAAACAAGCAGGGC -3′ | 296 |
| GDNF | F: 5′- GCAGCCGAAACAATGTACGA -3′  R: 5′- AAGGCGATGGGTCTGCAA -3′ | 100 |
| SCF | F: 5′- ATTGGTGGCAAATCTTCCCA -3′  R: 5′- TGCACTCCACAAGGTCATCAA -3′ | 217 |
| PDGFR-α | F: 5′- GGGAAGTTAGGGACAACTCGG -3′  R: 5′- GCTGGCAGAGGATTAGGCTC -3′ | 128 |
| P450scc | F: 5′- GCTAGCATCAAGGAGACGCT -3′  R: 5′- CCACCTGGTTGGGTCAAACT -3′ | 186 |
| STAR | F: 5′- TGGAAGTCCCTCAAGGACCA -3′  R: 5′- CACCTTGTCCCACTGTCCTC -3′ | 285 |
