# Supplementary material for: Analysis of Long Non-Coding RNA and mRNA Expression Profiling in Immature and Mature Bovine (Bos taurus) Testes
Source: Front Genet. 2019 Jul 5;10:646. doi: 10.3389/fgene.2019.00646 (PMC6624472; doi:10.3389/fgene.2019.00646)
Supplement: Supplementary file 15 [file Table_15.docx]

| Samples | Volume of ejaculate (mL) | Fresh sperm motility | Sperm concentration (10^8^/mL) |
| --- | --- | --- | --- |
| M1 | 5.54 ± 0.37 | 0.66 ± 0.16 | 11.08 ± 0.55 |
| M2 | 5.47 ± 0.45 | 0.63 ± 0.21 | 10.82 ± 0.57 |
| M3 | 5.94 ± 0.48 | 0.68 ± 0.13 | 11.62 ± 0.50 |

Table S15 Semen Quality Parameters of three mature Angus bulls.
